# Dapsone Hypersensitivity Syndrome With Leukemoid Reaction and Severe Thrombocytosis

**DOI:** 10.7759/cureus.14026

**Published:** 2021-03-21

**Authors:** Hans Raj Pahadiya, Manoj Lakhotia

**Affiliations:** 1 Medicine, Sawai Man Singh (SMS) Hospital, Jaipur, IND; 2 Internal Medicine, Dr. Sampurnanand Medical College, Jodhpur, IND

**Keywords:** dhs, leukemoid reaction, thrombocytosis

## Abstract

Hypersensitivity reactions to dapsone are common and potentially fatal adverse drug reactions. Herein, we report a case of a 45-year-old female who presented with fever, generalized desquamating rashes, and icterus three weeks after initiation of dapsone therapy for leprosy neuritis. She was diagnosed to have dapsone hypersensitivity syndrome (DHS) with leukemoid reaction and thrombocytosis. Dapsone was immediately discontinued, and she has treated with prednisolone 50 mg daily for a month and tapered over the next month. She has improved completely. DHS, leukemoid reaction, and severe thrombocytosis, or these adverse drug reactions to dapsone have rarely been reported.

## Introduction

Dapsone (4, 4’-diamino-diphenyl sulfone) is a potent anti-inflammatory and antibacterial compound. It is widely used in the treatment of leprosy and several chronic inflammatory dermatological conditions, such as dermatitis herpetiformis, chronic bullous dermatosis, Pneumocystis carinii [1]. Dapsone is also used as a second-line drug in the treatment of chronic immune thrombocytopenic purpura (ITP) to increase the platelets count [2]. The adverse effects of dapsone are common and include dose-related and idiosyncratic reactions. Dapsone hypersensitivity syndrome (DHS) was first reported by Lowe and was so named by Allday and Barnes [3,4]. DHS is a rare idiosyncratic reaction of the drug because of hypersensitivity and a life-threatening condition usually after initiation of dapsone therapy [5]. Herein, we described a case of DHS who initially presented with fever, icterus, and generalized rashes three weeks after initiation of dapsone therapy for leprosy neuritis. Our patient had a leukemoid reaction and severe thrombocytosis along with DHS which makes this case noteworthy. She was treated successfully with steroids.

## Case presentation

A 45-year-old non-alcoholic female presented with a seven-day history of fever, generalized rashes, deep jaundice, and itching three weeks after initiation of dapsone 100 mg/day for leprosy neuritis. At the time of admission, she was conscious, cooperative, and oriented. The blood pressure was 110/70 mmHg, pulse rate 110 beats/min, respiratory rate 16 breaths/min, and the temperature was 101.5 degrees Fahrenheit. She had an oral ulcer, deep icterus, pallor, and erythematosus, desquamating rashes predominantly over the neck, trunk, and upper extremities. The liver and spleen were not palpable. Cardiac and respiratory system examinations were unremarkable.

Laboratory investigations revealed a hemoglobin level of 7.0 g/dL, leukocyte count 79,500/mm^3^ with differential blood counts of neutrophils 75%, lymphocytes 3%, eosinophils 1%, myelocytes 13%, meta-myelocytes 06%, and a severe thrombocytosis of 14,32000/mm^3^. Peripheral blood film examination showed microcytic hypochromic anemia, leukocytosis with a shift to the left, and thrombocytosis. Liver function tests revealed total bilirubin of 12.14 mg/dL with direct fraction 7.5 mg/dL, aspartate transaminase (AST) 148 IU/L, alanine transaminase (ALT) 189 IU/L, alkaline phosphatase 622 IU/L, and total protein with serum albumin 2.8 g/dL. Lactate dehydrogenase (LDH) was markedly elevated at 1830 IU/L. Blood urea was 45 mg/dL, serum creatinine 0.75 mg/dL, serum sodium 138 mEq/L, and serum potassium 3.4 mEq/L. Urinalysis was normal. Coagulation profiles including bleeding time, clotting time, prothrombin time, and activated prothrombin time were within normal limits. Serological tests for hepatitis A virus (anti-HAV IgM), hepatitis B virus (HBsAg), hepatitis C virus (anti-HCV), and hepatitis E virus (anti-HEV IgM) were negative. She tested negative for malaria, leptospirosis, typhus fever, cytomegalovirus, Epstein-Barr virus, and human immunodeficiency virus 1 and 2. Other laboratory tests including thyroid hormone profile, glucose-6-phosphate dehydrogenase level, and iron profile were within normal limits. The direct, as well as indirect agglutination tests, were negative. The serum ferritin level was 285 ng/mL, leukocyte alkaline phosphatase (LAP) score was 205, erythrocyte sedimentation rate (ESR) 55/hr at the first hour, and C- reactive protein >6 mg/dl. In autoimmune study antinuclear antibody, anti-mitochondrial antibodies, anti-smooth muscle antibody, anti-double-stranded DNA, and anti-liver-kidney-microsomal antibodies were negative. Serial laboratory investigations are mentioned in Table 1. Chest X-ray and electrocardiogram were unremarkable. Ultrasonography of the abdomen showed mild uniform enlargement of the liver with normal echo-texture, there was no evidence of biliary obstruction and portal hypertension. Microscopic examination of the liver biopsy showed diffuse fatty changes and steatosis. Bile pigment in hepatocytes and bile in bile canaliculi was present. The mild proliferation of the bile duct and mild inflammation of the portal triad were also present.

**Table 1:** Laboratory Investigations Hb- hemoglobin, TLC- total leukocytes count, DLC- differential leukocytes count, N- neutrophils, L- lymphocytes, M- monocyte, E- eosinophils, AST- aspartate aminotransferase, ALT- alanine aminotransferase, LDH- lactate dehydrogenase, ESR- erythrocyte sedimentation rate, CRP- C-reactive protein,  Alk P- alkaline phosphatase,  LAP score- leukocyte alkaline phosphate score

Laboratory investigations							
Date/parameters	16.9.14	08.12.14	09.12.14	12.12.14	16.12.14	18.12.14	25.12.14
Hb (g/dL)	11.99	7.1	7.0	7.3	8.1	8.2	10.2
TLC (cells/mm^3^)	6170	68,200	79500	32560	25630	17360	9800
DLC (N/L/M/E/) %	65/29/5/1		75/3/1/1	86/8.4/3.5/2	77.5/15.7/6.4	78.5/13.5/7.5/0.3	75/18/5
Platelets (cells/mm^3^)	3,68000	13,62000	14,32000	6,68000	5,34000	5,13000	3,12000
B. Sugar (g/dL)			88	90	67		
B. Urea (g/dL)			45	50	23		
S. Creat (g/dL)			0.75	0.81	0.65		
S. Bil T (g/dL)			12.14	15.5	17.3	14.4	
D (g/dL)			7.5	10.45	9.8	6.31	
AST (IU/L)			189	229	122	155	
ALP (IU/L)			148	195	136	142	
S. Albumin (g/dL)			2.8	3.2	3.1		
Alk P (IU/L)			622	642	650	442	
LDH (IU/L)			1830	1620	635		
LAP score			205	178	165		65
ESR			55/1st hr	54/1st hr			
CRP (g/dL)			>6	>6	>6	>6	
S. Ferritin (ng/L)			285				

Clinical history of the patient and laboratory parameters with a prior history of dapsone therapy was consistent with a diagnosis of DHS with leukemoid reaction and thrombocytosis. She was treated with oral prednisolone 50 mg/day (1 mg/kg) for one month and then tapered off over the next month. She started improving after the first week with gradual subsidence of fever and rashes. Leukocytes, platelet count, and leukocyte alkaline phosphate (LAP) score showed a downward trend on repeated examination (Table 1). After one month of follow-up, she was asymptomatic with complete resolution of rashes, icterus, with normal hematological parameters.

## Discussion

The adverse effect of dapsone, a generalized hypersensitivity reaction is termed DHS. This occurs usually after an interval of three to five weeks from the initiation of therapy in most cases. This syndrome may begin as early as seven to 10 days after administration of the drug to as late as six months into therapy with dapsone [6,7]. The most common signs and symptoms of DHS are sudden onset of fever, followed by generalized rashes, hepatitis, lymphadenopathy, nausea and vomiting, eosinophilia, and mucosal involvement in decreasing order [6]. Other manifestations are hepatitis, hemolysis, agranulocytosis, methemoglobinemia, lymphocytosis, and toxic epidermal necrolysis. The Stevens-Johnson syndrome, agammaglobulinemia, raised ESR, pneumonitis, carditis, nephritis, and leukemoid reaction are rarely reported. Hemolysis and hepatotoxicity both can lead to hyperbilirubinemia in dapsone syndrome [7,8].

In hepatic dysfunctions, hepatocellular injury is less common than cholestatic injury. Hepatocellular injury is more severe, characterized by elevated transaminases with liver biopsy showing predominantly eosinophilic lobular and portal infiltration whereas, cholestatic injury is less severe with high alkaline phosphatase level, modest rise in transaminases level, and may have granuloma on biopsy. The mechanism of injury, including hepatotoxicity in dapsone syndrome, seems to be a hypersensitivity reaction [9]. The incidence of DHS was found 1.4% with an overall case-fatality of 9.9% in a review [6]. The outcome of DHS depends on mucosal involvement, rash, hepatitis, older age, leprosy as an indication for dapsone, and living in non-affluent countries [6]. The mechanism of DHS is unclear. Knowles SR et al. considered this as a combination of type I, type IV, and perhaps type III Gel and Coombs hypersensitivity reactions and alternatively modified graft versus host disease mediated by activated T-lymphocytes [10].

The metabolic differences in the production and detoxification of intermediate products of dapsone metabolism play an important role in the pathogenesis of sulfonamide hypersensitivity reactions [11]. Dapsone is metabolized in the liver primarily via N-acetylation and N-hydroxylation pathway [1,11]. The intermediate product of the N-hydroxylation pathway such as nitrosamines and possibly other compounds such as responsible for side effects of the drug. The generation of free radicals by dapsone hydroxylamine and subsequent depletion of red blood cell glutathione stores are responsible for hemolysis and the reaction of dapsone hydroxylamine with oxyhemoglobin leads to methemoglobinemia [12,13]. Agranulocytosis appears due to decreased production of granulocytes in bone marrow because of involved cell control mechanisms as opposed to toxicity or an immunologic reaction [14]. The activity of the N-hydroxylation pathway in the liver depends on the individual, genetic factor (cytochrome P450 activity, decreased reduced glutathione), and environmental factors. There is decreased production of toxic metabolites in old age patients and patients with pre-existing liver disease because of decreased enzyme activity, hence there are fewer chances of adverse events of dapsone in these candidates [11].

Dapsone is also used as a second-line drug in the treatment for chronic and refractory ITP to increase platelets. The underlying mechanism for the increase in the platelet count is unclear. A case of thrombocytosis in a patient of HIV with Pneumocystis jirovecii infection during treatment with dapsone has been reported [15]. The mechanism for this is explained by interference with the reticuloendothelial system brought about by increased erythro-phagocytosis secondary to the excessive red blood cell destruction induced by dapsone [16]. Dapsone also competitively inhibits the myeloperoxidase system and interferes with phagocyte-mediated cytotoxicity [17].

Our patients presented with mucocutaneous symptoms, fever, and icterus because of a predominantly cholestatic pattern of hepatic injury. She also had a leukemoid reaction and thrombocytosis. Leukemoid reaction in our case may be secondary to dapsone-triggered hemolysis which is supported by anemia, hyperbilirubinemia, and increased level of LDH. An increase in the leukocyte counts occurs in hemolytic anemia because of compensatory bone marrow response. On the other hand, thrombocytosis is supposed to be reactive as our patient had increases levels of ESR, C-reactive protein (CRP), and ferritin or this can be supported by the role of dapsone in ITP to increase platelets, but such a marked increased in platelets count has not been reported in follow up of ITP treatment with dapsone.

## Conclusions

We conclude that clinicians should be vigilant about the possibility of leukemoid reaction and thrombocytosis as a part of hypersensitivity reaction to dapsone. Usually, DHS is a self-limiting reaction after cessation of dapsone, but the early withdrawal of the drug, symptomatic treatment, and systemic corticosteroids should be used to improve the outcome of DHS. In a patient who had a dapsone hypersensitivity reaction with leukocytosis, the leukemoid reaction should be considered as a differential diagnosis. Patients who develop DHS should avoid dapsone in the future. Here, our case report is unique due to an underappreciated combination of DHS with leukemoid reaction and thrombocytosis because of hemolytic effect of dapsone. Thrombocytosis might be due to the stimulatory effect of dapsone on the platelet. 
